# The performance of different case definitions for severe influenza surveillance among HIV-infected and HIV-uninfected children aged <5 years in South Africa, 2011–2015

**DOI:** 10.1371/journal.pone.0222294

**Published:** 2019-09-19

**Authors:** Hetani Ngobeni, Stefano Tempia, Adam L. Cohen, Sibongile Walaza, Lazarus Kuonza, Alfred Musekiwa, Anne von Gottberg, Orienka Hellferscee, Nicole Wolter, Florette K. Treurnicht, Jocelyn Moyes, Fathima Naby, Omphile Mekgoe, Cheryl Cohen

**Affiliations:** 1 South African Field Epidemiology Training Programme, Johannesburg, South Africa; 2 School of Health Systems and Public Health, University of Pretoria, Pretoria, South Africa; 3 Centre for Respiratory Diseases and Meningitis, National Institute for Communicable Diseases of the National Health Laboratory Service, Johannesburg, South Africa; 4 Influenza Division, Centers for Disease Control and Prevention, Atlanta, Georgia, United States of America; 5 Influenza Program, Centers for Disease Control and Prevention, Pretoria, South Africa; 6 MassGenics, Duluth, Georgia, United States of America; 7 Global Immunization Monitoring and Surveillance Team, Vaccines and Biologicals, World Health Organisation, Geneva, Switzerland; 8 School of Public Health, University of the Witwatersrand, Johannesburg, South Africa; 9 School of Pathology, Faculty of Health Sciences, University of the Witwatersrand, Johannesburg, South Africa; The University of Hong Kong, CHINA

## Abstract

In 2014, the World Health Organization (WHO) proposed a new severe influenza surveillance case definition, which has not been evaluated in a high human immunodeficiency virus (HIV) prevalence setting. Our study aimed to assess the performance of this proposed case definition in identifying influenza among HIV-uninfected and HIV-infected children aged <5 years in South Africa. We prospectively enrolled children aged <5 years hospitalised with physician-diagnosed lower respiratory tract infection (LRTI) at two surveillance sites from January 2011 to December 2015. Epidemiologic and clinical data were collected. We tested nasopharyngeal aspirates for influenza using reverse transcription polymerase chain reaction. We used logistic regression to assess factors associated with influenza positivity among HIV-infected and HIV-uninfected children. We calculated sensitivity and specificity for different signs and symptoms and combinations of these for laboratory-confirmed influenza. We enrolled 2,582 children <5 years of age with LRTI of whom 87% (2,257) had influenza and HIV results, of these 14% (318) were HIV-infected. The influenza detection rate was 5% (104/1,939) in HIV-uninfected and 5% (16/318) in HIV-infected children. Children with measured fever (≥38°C) were two times more likely to test positive for influenza than those without measured fever among the HIV-uninfected (OR 2.2, 95% Confidence Interval (CI) 1.5–3.4; p<0.001). No significant association was observed between fever and influenza infection among HIV-infected children. Cough alone had sensitivity of 95% (95% CI 89–98%) in HIV-uninfected and of 100% (95% CI 79–100%) in HIV-infected children but low specificity: 7% (95% CI 6–8%) and 6% (95% CI 3–9%) in HIV-uninfected and HIV-infected children, respectively. The WHO post-2014 case definition for severe acute respiratory illness (SARI—*an acute respiratory infection with history of fever or measured fever of ≥ 38°C and cough; with onset within the last ten days and requires hospitalization*), had a sensitivity of 66% (95% CI 56–76%) and specificity of 46% (95% CI 44–48%) among HIV-uninfected and a sensitivity of 63% (95% CI 35–84%) and a specificity of 42% (95% CI 36–48%) among HIV-infected children. The sensitivity and specificity of the WHO post-2014 case definition for SARI were similar among HIV-uninfected and HIV-infected children. Our findings support the adoption of the 2014 WHO case definition for children aged <5 years irrespective of HIV infection status.

## Introduction

Influenza infection causes an estimated 291,243–645,832 respiratory deaths globally each year [1]. Children aged<5 years have an increased risk of influenza-associated hospitalisation compared to older individuals [2]. Human immunodeficiency virus (HIV)-infected children are at a higher risk of severe influenza-associated illness and death compared to HIV-uninfected children [3][4]. The overall HIV prevalence among children aged 0–14 years in South Africa was 2.4% in 2012[5]. The Antiretroviral Therapy (ART) coverage among South African HIV-infected children was 47.4% in 2015 [6]. HIV-infected children hospitalised with influenza-associated lower respiratory tract infection (LRTI) often have a more severe clinical course compared to HIV–uninfected children[5]. During 2010–2012, the World Health Organization (WHO), following a consultation process, changed the recommended influenza surveillance case definition for severe acute respiratory illness (SARI) to any child with an acute respiratory infection with history of fever or measured fever of ≥ 38°C and cough; with onset within the last ten days and requires hospitalization.

The balancing act of satisfying two potentially differing needs of a surveillance case definition, sensitivity versus specificity, largely depends on the surveillance system objectives. A case definition that is highly sensitive will capture a higher proportion of the total cases, but will also increase the number of persons tested. While a highly specific case definition will capture a higher proportion of true positives, a smaller proportion of all cases will be identified [7][8]. It has been shown that the presence of cough and fever in a patient is likely to be indicative of influenza; however, more than half of symptomatic children with influenza may not have a documented fever[8][9]. In addition, it is not clear whether HIV-infected children with influenza-associated LRTI have a different clinical presentation compared to HIV-uninfected children.

The 2014 WHO case definition was developed for global influenza surveillance use; however, its performance in a high HIV prevalence setting is unknown. In this study we evaluated the sensitivity, specificity and predictive values of the 2014 WHO SARI surveillance case definition as well as a range of signs and symptoms in identifying influenza virus infections among HIV-uninfected and HIV-infected children aged <5 years hospitalised at two surveillance hospitals in South Africa.

## Materials and methods

### Description of the surveillance program

The severe acute respiratory illness (SARI) surveillance program was initiated in 2009 as an active, prospective, hospital-based surveillance with sites in five of the nine provinces in South Africa. Our analysis was limited to patients enrolled at two sites, namely; Edendale Hospital and Klerksdorp-Tshepong Hospital Complex. The Edendale Hospital is situated in a peri-urban area of KwaZulu-Natal Province while the Klerksdorp- Tshepong Hospital Complex is in a peri-urban area of the North West Province.

At these two sentinel sites, hospitalised children who met an expanded surveillance case definition were prospectively enrolled. The case definition included children aged <5 years with physician-diagnosed LRTI irrespective of symptoms and symptom duration as well as children with suspected or confirmed tuberculosis. In addition children aged 2 days to <3 months with suspected sepsis were also enrolled.

### Study population and procedures

Our study population consisted of all children aged <5 years, enrolled into the SARI programme using the above mentioned expanded case definition at Edendale and Klerksdorp hospitals, between January 2011 and December 2015. Hospitalised individuals were screened by trained surveillance officers to assess enrolment eligibility. Parents or guardians of children who met the surveillance case definitions were approached for study enrolment. A case investigation form including clinical details and follow up for in-hospital outcome was completed for all enrolled patients using a combination of interview and medical records review.

### Laboratory methods

Nasopharyngeal aspirates (NPA) were collected from enrolled patients and transported in universal transport medium at 4–8°C to the National Institute for Communicable Diseases (NICD) within 72 hours of collection for testing. NPA were tested for influenza A and B viruses as well other respiratory viruses, using an in-house multiplex real-time reverse-transcription polymerase chain reaction (RT-PCR) from 2011 through 2014 and the commercial Allplex Respiratory Assay (panel 2 and 3) (Seegene, Seoul, Korea) in 2015 [10]. Influenza A and B and respiratory syncytial viruses were tested using the commercial FTD Flu/RSV assay (Fast Track Diagnostics, Luxembourg) in 2015. Data from case investigation forms (CIF) and laboratory results were entered in a centralised database maintained at the NICD.

### Determination of HIV status

The determination of HIV status in consenting patients was done as part of standard-of-care or through dried blood spot specimen testing, by HIV PCR for children <18 months of age and by ELISA for children >18 months of age [11]. CD4+ T-cell counts were determined by flow cytometry[12]. HIV exposed but uninfected children at the time of enrolment were considered HIV-uninfected in this analysis.

### Statistical analysis

Patients with missing HIV status or influenza results were excluded from the analysis. We implemented two analyses to assess: (i) factors associated with HIV infection among influenza-positive children; and (ii) factors associated with influenza positivity among HIV-infected and HIV-uninfected children separately. For both analyses, we used unconditional logistic regression. We used exact logistic regression for variables where there were counts of 0. All factors with p<0.2 on univariate analysis were assessed for significance in the multivariable model. Only variables with p<0.05 were kept in the final multivariable model through manual backward elimination. The fit of the multivariable models was assessed using the Hosmer-Lemeshow goodness-of-fit test. We included ‘study site’ in the model *a priori* to control for potential differences by study location. In addition, we calculated the sensitivity, specificity, positive predictive values (PPV) and negative predictive values (NPV) for individual symptoms and selected case definitions reported in Table 1. This analysis was implemented in the overall sample and stratified by HIV status. All analyses were performed with Stata 13 (StataCorp, College Station, TX, USA).

**Table 1:** WHO surveillance case definitions evaluated.

Case definition	Criteria
WHO before 2011	Any child aged 2 months to 5 years with cough or difficulty in breathing *and* unable to drink or breastfeed, or vomits everything, or convulsions, or lethargic or unconscious, or chest indrawing or stridor in a calm child and requires hospital admission [13].
WHO 2014 (official)	An acute respiratory infection with history of fever or measured fever of ≥ 38°C and cough; with onset within the last ten days and requires hospitalization [14].
WHO 2014 (with fever of ≥ 38°C and cough only)	An acute respiratory infection with measured fever of ≥ 38°C and cough; with onset within the last ten days and requires hospitalization.

### Ethical considerations

The SARI surveillance protocol was approved by the Human Research Ethics Committee of the University of the Witwatersrand (M081042) and the University of KwaZulu-Natal Ethics Committee (BF157/08). Additional ethical clearance to conduct the analysis was sought from the Faculty of Health Sciences Research Ethics Committee of the University of Pretoria (No. 429/2014). This project was reviewed in accordance with CDC human research protection procedures and was determined to be non-research, (routine disease surveillance activity; non-research determination number: 2012–6197). Informed consent for data collection (both interviews and medical record reviews), and laboratory testing, and use of data for research was obtained from legal guardians. Collected surveillance data were anonymized before accessed for this analysis.

## Results

### Study population and influenza virus detection

**Fig 1:**
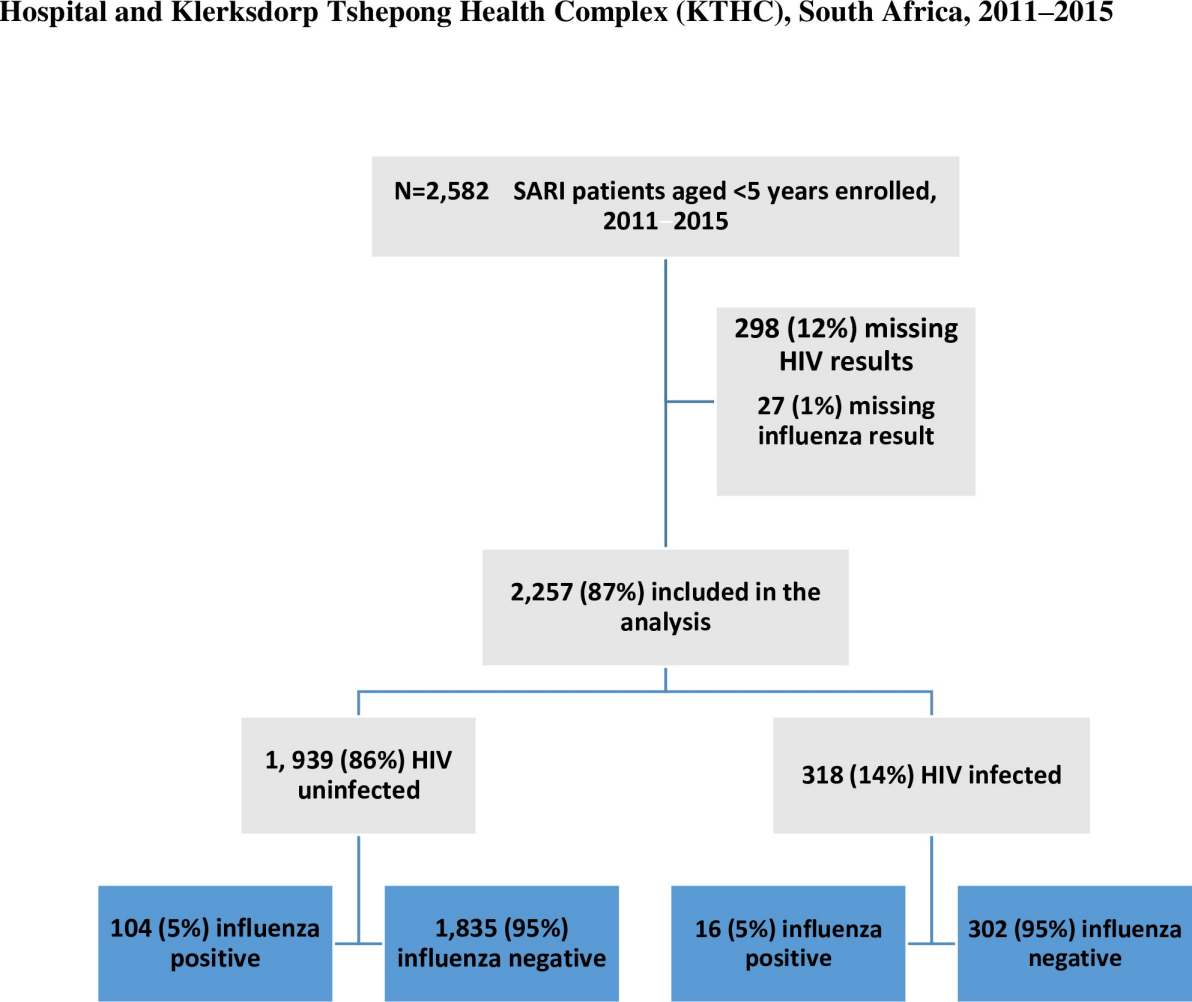
Flow diagram showing cohort of children aged < 5years, South Africa, January 2011 to December 2015.

During January 2011-December 2015, we enrolled 2,582 children aged <5 years (Fig 1). We excluded 298 (12%) children who had no HIV result and 27 (1%) children who had missing influenza results. The demographic characteristics among those with HIV and influenza results were similar to those with missing results in terms of age, sex and race. Influenza results were missing for various reasons including insufficient specimen. Among the 2,257 children included in our analysis, 318 (14%) were HIV-infected. Overall influenza viruses were detected in specimens from 5.3% (120/2,257) of children with test results, in 5.4% (104/1939) of HIV-uninfected children and 5.0% (16/318) of HIV-infected children.

### Factors associated with HIV infection and influenza virus positivity

In the analysis of factors associated with HIV-infection, we found no statistically significant differences for the investigated epidemiological and clinical characteristics between HIV-infected and HIV-uninfected children that tested positive for influenza viruses (Table 2).

**Table 2:** Epidemiological and clinical characteristics of HIV-infected and HIV-uninfected children aged <5 years testing influenza positive and hospitalised with severe respiratory illness at the Edendale and Klerksdorp hospitals, South Africa, 2011–2015.

		All patients	HIV-infected	HIV-uninfected	Unadjusted	
Characteristic						p-value
		n/N (%)	n/N (%)	n/N (%)	OR (95% CI*)	
**Age(y)**	<1	57/120 (48)	6/16 (38)	51/104 (49)	Reference	
	1–4	63/120 (53)	10/16 (63)	53/104 (51)	1.6 (0.5–5.7)	0.39
**Sex**	Male	73/120 (61)	9/16 (57)	64/104 (62)	1.2 (0.4–3.0)	0.69
**Race**	Black	114/115 (99)	16/16 (100)	98/99 (99)	6 (0–241)	1
						
**Year**	2011	33/120 (28)	7 /16 (43)	26/104 (25)	Reference	
	2012	30/120 (25)	3/16 (19)	27/104 (26)	0.4 (0.1–2.1)	0.22
	2013	36/120 (30)	4/16 (25)	27/104 (31)	0.6 (0.1–2.5)	0.38
	2014	20/120 (17)	2/16 (13)	32/104 (17)	0.2 (0.1–1.4)	0.30
	2015	1/120 (1)	0/16 (0)	1/104 (1)	Empty	
**Site**	KTHC	51/120 (43)	10/16 (62)	41/104 (39)	2.6 (0.9–7.8)	0.09
**Clinical presentation**						
	Any fever	87/120 (73)	10/16 (63)	77/104 (74)	0.6 (0.2–1.8)	0.34
	Fever ≥38*°*C	63/120 (53)	7/16 (44)	56/104 (54)	0.7 (0.2–1.9)	0.45
	Cough	114/119 (96)	16/16 (100)	98/103 (95)	1 (0.1–inf)	0.95
	Difficulty breathing	73/119 (61)	9/16 (56)	64/103 (62)	0.8 (0.3–2.3)	0.65
	Diarrhoea	25/119 (21)	6/16 (38)	19/103 (18)	2.7 (0.9–8.2)	0.09
	Vomiting	9/119 (8)	3/16 (19)	6/103 (6)	3.7 (0.8–17)	0.08
	Inability to feed	21/119 (18)	2/16 (13)	19/103 (18)	0.6 (0.1–3.0)	0.56
	Lethargy	49/119 (41)	7/16 (44)	42/103 (41)	1.1 (0.4–3.2)	0.82
	Convulsions	10/119 (8)	1/16 (6)	9/103 (9)	0.7 (0.08–5.9)	0.74
	Stridor	21/119 (18)	4/16 (25)	17/103 (17)	1.7 (0.5–5.9)	0.41
**Indicators for disease severity**						
	Ventilation	61/118 (52)	8/16 (50)	53/102 (52)	0.9 (0.3–2.7)	0.88
	Death	1/118 (0.8)	0/16 (0)	1/102 (1)	6 (0–249)	1.00

*CI: Confidence Interval.

In the analysis of factors associated with influenza virus detection among HIV-uninfected children, only measured fever ≥38°C (aOR = 2.2, 95% CI 1.5–3.4) was significantly more common amongst influenza-positive children in the multivariable analysis (Table 3). Among HIV-infected children no signs or symptoms were significantly associated with testing influenza positive but numbers of influenza positive children were low (16) (Table 3).

**Table 3:** Factors associated with influenza virus positivity among HIV-uninfected and HIV-infected children aged <5 years hospitalised with severe respiratory illness at the Edendale and Klerksdorp hospitals, South Africa, 2011–2015.

		HIV-uninfected					HIV-infected		
Symptom		Influenza positive n/N (%)	Unadjusted OR (95%CI*)	p-value	Adjusted OR (95%CI*)	p-value	Influenza positive n/N (%)	Unadjusted OR (95%CI*)	p-value
									
**Fever (> = 38**°C**)**	N	48/1 260 (4)	Ref				9/176 (5)	Ref	
** **	Y	56/679 (8)	2.3 (1.5–3.4)	<0.001	2.2 (1.5–3.4)	<0.001	7/142(5)	0.9 (0.3–2.7)	0.94
**Any fever**	N	27/738 (4)	Ref				6/102 (6)	Ref	
** **	Y	77/1 194 (6)	1.8 (1.1–2.8)	0.007			10/212 (5)	0.8 (0.2–2.2)	0.66
**Cough**	N	5/136 (4)	Ref				0/17 (0)	Ref	
	Y	98/1 795 (5)	1.5 (0.6–3.7)	0.37			16/300 (5)	1.3 (0.2– +inf)	0.80
**Symptoms duration (days)**									
** **	≤7	88/1 754 (5)	Ref				12/264 (5)	Ref	
** **	8–10	3/ 32 (9)	2 (0.5–6.5)	0.26			1/7 (14)	3.5 (0.3–31.4)	0.26
** **	11–14	2/ 26 (8)	1.6 (0.3–6.7)	0.54			1/10 (10)	2.3 (0.2–20)	0.43
** **	15+	5/ 66 (8)	1.6 (0.6–3.9)	0.36			2/21 (10)	2.2 (0.4–11)	0.32
**Vomiting**	N	97/1 844 (5)	Ref				13/295 (4)	Ref	
** **	Y	6/87 (7)	1.3 (0.6–3.1)	0.50			3/22 (13)	3.4 (0.9–13)	0.07
**Diarrhoea**	N	84/1 671 (5)	Ref				10/245 (4)	Ref	
** **	Y	19/260 (7)	1.5 (0.9–2.5)	0.13			6/72 (8)	2.1 (0.7–6)	0.15
**Inability to feed**	N	84/1 637 (5)	Ref				14/270 (5)	Ref	
** **	Y	19/295 (6)	1.3 (0.8–2.1)	0.36			2/47 (4)	0.8 (0.1–3.7)	0.79
**Lethargy**	N	61/1 098 (6)	Ref				9/156 (6)	Ref	
** **	Y	42/832 (5)	0.9 (0.6–1.4)	0.62			7/161 (4)	0.7 (0.3–2.0)	0.57
**Convulsions**	N	94/1 856 (5)	Ref				15/311 (5)	Ref	
** **	Y	9/75 (12)	2.6 (1.2–5.3)	0.02			1/6 (17)	4 (0.4–36)	0.22
**Stridor**	N	86/1 426 (6)	Ref				12/242 (5)	Ref	
** **	Y	17/505 (3)	0.5 (0.3–0.9)	0.02			4/75 (3)	0.1 (0.03–3.4)	0.90
**Difficulty breathing**	N	39/612 (6)	Ref				7/109 (10)	Ref	
	Y	64/1 320 (4)	0.7 (0.5–1.1)	0.17			9/208 (4)	0.7 (0.2–1.8)	0.42
**Site**	Eden	63/1 272 (5)	Ref				6/167 (4)	Ref	
	KTHC	41/667 (6)	1.3 (0.8–1.9)	0.26	1.7 (0.9–1.3)	0.54	10/151 (7)	1.9 (0.7–5.4)	0.22

*CI: Confidence Interval

Hosmer-Lemeshow Goodness-of-fit-test for the multivariable model among HIV-uninfected children, fever (> = = 38°C) & site: p-value 0.91.

### Sensitivity and specificity of individual signs and symptoms

Among HIV–infected and HIV–uninfected children aged <5 years combined, individual symptoms for influenza virus positivity with high sensitivity included any fever (72%; 95% CI 63–80%) and cough (96%; 95% CI 91–99%). Diarrhoea (86%; 95% CI 84–87%), vomiting (95%; 95% CI 94–96%), inability to feed (85%; 95% CI 83–86%), and convulsions 97% (95% CI 96–98%) had high specificity. The sensitivity of duration of symptom of ≤7, ≤10 or ≤14 days was similar (range: 91–92%). Using a symptom duration cut-off of ≤7 days, 100/114 (88%) children would have been enrolled; 4 (104/114; 91%) additional children had symptom duration ≤10 days, 3 (107/114; 94%) additional children had symptom duration ≤14 days and 7 (114/114; 100%) additional children had symptom duration ≥15 days (Table 4).

**Table 4:** Sensitivity, specificity and predictive values of signs and symptoms for influenza virus positivity among HIV-infected and HIV-uninfected children aged <5 years hospitalised with severe respiratory illness at the Edendale and Klerksdorp hospitals, South Africa, 2011–2015.

Factors	Influenza positivity		Sensitivity	Specificity	PPV*	NPV**
	Presence of factor n/N (%)	Absence of factor n/N (%)				
**Individual clinical signs and symptoms (n = 2 257)**						
Fever >38°C	63/821 (8)	57/1 436 (3)	53 (43–62)	64 (62–67)	8 (6–10)	96 (94–97)
Any fever	87/1 406 (6)	33/840 (4)	73 (63–80)	38 (36–40)	6 (5–8)	96 (95–97)
Cough	114/2 095 (5)	5/153 (3)	96 (91–99)	7 (6–9)	5 (5–7)	97 (93–99)
Difficulty breathing	73/1 528 (5)	46/721 (6)	61 (52–70)	32 (28–34)	5 (4–6)	94 (92–95)
Diarrhoea	25/332 (8)	94/1 916 (5)	21 (14–30)	86 (84–87)	8 (5–11)	95 (94–96)
Vomiting	9/109 (8)	110/2 139 (5)	8 (4–14)	95 (94–96)	8 (3–15)	95 (94–96)
No feeding	21/342 (6)	98/1 907 (5)	18 (11–26)	85 (83–86)	6 (3–8)	94 (93–96)
Lethargy	49/993 (5)	70/1 254 (6)	41 (32–51)	56 (53–58)	5 (3–7)	94 (93–96)
Convulsion	10/81 (12)	109/2 167 (5)	8 (4–12)	97 (96–98)	12 (6–22)	95 (94–96)
Stridor	21/580 (4)	98/1 668 (6)	18 (11–26)	74 (71–76)	4 (2–6)	94 (93–95)
**Symptoms duration in days (n = 1 774)**						
≤7 days	14/162 (9)	100/2 018 (5)	91 (86–95)	5 (4–6)	7 (6–8)	88 (82–93)
≤10 days	10/123 (8)	104/2 057 (5)	92 (86–96)	5 (4–6)	6 (5–7)	91 (85–96)
≤14 days	7/87 (8)	107/2 093 (5)	92 (84–97)	5 (4–6)	4 (3–5)	94 (88–96)
**Number of influenza-positive patient (n = 114)**			**No. cases**	**Cumulative number**	**Cumulative %**	
≤7 days			100	100	88	
≤10 days			4	104	91	
≤14 days			3	107	94	
15+ days			7	114	100	

*PPV: positive predictive value.

**NPV: negative predictive value.

Among HIV-uninfected children, measured fever (54%; 95% CI 43–64%), any fever (74%; 95% CI 64–82%), cough (95%; 95% CI 89–98%) and difficulty breathing (62%; 95% CI 52–72%) had high sensitivity for influenza positivity; whereas diarrhoea (87%; 95% CI 85–88%), vomiting (96%; 95% CI 95–97%) and inability to feed (85%; 95% CI 83–87%) had high specificities (Table 5).

**Table 5:** Sensitivity, specificity and predictive values of signs and symptoms for influenza virus positivity among HIV-uninfected and HIV-infected children aged <5 years hospitalised with severe respiratory illness at the Edendale and Klerksdorp hospitals, South Africa, 2011–2015.

		Value (%) and 95% Confidence Interval						
HIV uninfected					HIV infected			
** **	**Sensitivity**	**Specificity**	PPV*	NPV**	**Sensitivity**	**Specificity**	PPV*	NPV**
**Individual clinical signs and symptoms**					** **			
**Fever >38**°C	54 (43–64)	66 (64–68)	8 (6–10)	96 (95–97)	44 (20–70)	55 (50–61)	5 (2–10)	95 (91–98)
**Any fever**	74 (64–82)	39 (37–41)	6 (5–8)	96 (95–98)	63 (27–85)	32 (27–39)	5 (2–9)	194 (88–98)
**Cough**	95 (89–98)	7 (6–8)	6 (5–7)	96 (92–99)	100 (79–100)	6 (3–9)	5 (3–9)	100 (80–100)
**Difficulty breathing**	62 (52–72)	31 (29–33)	5 (4–6)	94 (91–95)	56 (30–80)	34 (29–40)	4 (2–8)	94 (87–97)
**Diarrhoea**	18 (11–27)	87 (85–88)	7 (5–11)	95 (94–96)	38 (15–65)	78 (73–83)	8 (3–17)	96 (93–98)
**Vomiting**	6 (2–12)	96 (95–97)	7 (3–11)	95 (94–96)	19 (4–47)	94 (90–96)	14 (3–35)	96 (93–98)
**No feeding**	18 (12–27)	85 (83–87)	6 (4–10)	95 (94–96)	13 (2–38)	85 (81–89)	4 (1–15)	95 (92–97)
**Lethargy**	41 (31–51)	57 (55–60)	5 (4–7)	94 (93–96)	44 (20–70)	49 (43–55)	4 (2–9)	94 (89–97)
**Convulsion**	9 (4–16)	96 (95–97)	12 (6–22)	95 (94–96)	6 (0.2–30)	98 (96–100)	17 (0.4–64)	95 (92–97)
**Stridor**	17 (10–25)	73 (71–75)	3 (2–6)	93 (94–95)	25 (7–52)	76 (71–81)	5 (2–13)	95 (92–97)
**Symptoms duration in days**								
≤**7**	92 (86–96)	5 (4–6)	6 (5–8)	90 (82–95)	90 (75–97)	6 (3–8)	12 (8–16)	75 (48–93)
≤**10**	92 (85–97)	5 (4–6)	5 (4–6)	93 (86–97)	90 (74–98)	5 (3–8)	10 (7–14)	81 (54–96)
≤**14**	92 (83–98)	5 (4–6)	3 (3–4)	95 (89–98)	91 (69–99)	5 (3–8)	7 (4–10)	88 (62–98)

*PPV: positive predictive value

**NPV: negative predictive value

Among HIV–infected children, measured fever (44%; 95% CI 20–70%), any fever (63%; 95% CI 27–85%) and cough (100%; 95% CI 79–100%) had moderate to high sensitivity. In the same group, diarrhoea (78%; 95% CI 73–83%), vomiting (94%; 95% CI 90–96%), inability to feed (85%; 95% CI 81–89%), convulsions (98%; 95% CI 96–100%) and stridor (76%; 95% CI 71–81%) yielded high specificities (Table 5). There were no significant differences in the sensitivity and specificity of different sign and symptoms between HIV-infected and HIV-uninfected children aged <5 years. Sensitivity remained constantly high and specificity remained constantly low irrespective of duration of symptoms among HIV-infected and HIV-uninfected children (Table 5).

### Sensitivity and specificity of selected case definitions

The pre-2011 WHO case definition had a sensitivity of 66% (95% CI 56–76%) and a specificity of 32% (95% CI 29–34%) among HIV-uninfected children and a sensitivity of 63% (95% CI 35–85%) and a specificity of 31% (95% CI 26–37%) among HIV-infected children. The 2014 WHO case definition had a sensitivity of 66% (95% CI 56–76%) and a specificity of 46% (95% CI 44–48%) among HIV-uninfected children and a sensitivity of 63% (95% CI 35–84%) and a specificity of 42% (95% CI 36–48%) among HIV-infected children (Table 6).

**Table 6:** Sensitivity, specificity and predictive values of various case definitions for influenza virus positivity among HIV-uninfected and HIV-infected children aged <5 years hospitalised with severe respiratory illness at the Edendale and Klerksdorp hospitals, South Africa, 2011–2015.

** **					Value (%) and 95% Confidence Interval							
HIV-uninfected					HIV-infected				All			
	Sensitivity	Specificity	PPV*	NPV**	Sensitivity	Specificity	PPV*	NPV**	Sensitivity	Specificity	PPV*	NPV**
**Various case definitions**												
2011WHO	66 (56–76)	32 (29–34)	6 (5–7)	94 (91–96)	63 (35–85)	31 (26–37)	5 (2–9)	94 (87–98)	66 (56–74)	32 (30–34)	6 (5–7)	94 (91–96)
2014WHO	66 (56–76)	46 (44–48)	6 (5–8)	96 (95–97)	63 (35–84)	42 (36–48)	6 (3–10)	95 (90–98)	66 (56–74)	45 (43–47)	6 (5–8)	96 (95–97)
2014WHO (fever≥38)#	48 (38–58)	70 (68–73)	8 (6–11)	96 (95–97)	44 (20–70)	61 (55–67)	6 (2–12)	95 (91–98)	47 (38–57)	69 (67–71)	8 (6–10)	96 (95–97)

*PPV: positive predictive value.

**NPV: negative predictive value

#Documented fever and cough.

Compared to the 2014 WHO case definition, the 2014 WHO case definition with fever ≥38°C only and cough had lower sensitivity, but higher specificity among both HIV-uninfected and HIV-infected children (Table 6).

## Discussion

We evaluated the pre-2011 and 2014 WHO SARI case definitions in HIV-infected and HIV-uninfected children in South Africa. Other studies have evaluated sensitivity, specificity and predictive values for influenza case definition [15][8]. However, to the best of our knowledge, this is the first study to evaluate sensitivity, specificity and predictive values for the pre-2011 and 2014 WHO SARI case definitions stratified by HIV status. We found that the 2014 WHO case definition performed similarly in both HIV-uninfected and HIV-infected children aged <5 years.

We found the clinical presentation of HIV-uninfected and HIV-infected children was similar among the factors studied. Fever of ≥38°C was associated with influenza positivity in HIV-uninfected children and this has also been reported in other studies that found measured fever as an accurate predictor of influenza infection [16][17]. We did not observe any significant association between fever and influenza infection in the HIV-infected children, possibly because numbers of HIV-infected influenza-positive children were low. However, the above assertion does not rule out the possibility that response to influenza could also be different in this group.

The inclusion of cough in the 2014 WHO case definition is supported by our findings of very high sensitivity; 95% and 100% among HIV-uninfected and HIV-infected children, although specificity was low in both groups. The presence of cough has been previously demonstrated to be a good predictor for influenza positivity [8][14].

In our setting, symptom duration (≤7, ≤10, or ≤14 days) did not impact sensitivity and specificity of the WHO case definition. An earlier publication using the same dataset showed that influenza positive children aged ≤5 years with duration of symptoms ≤7 days accounted for 79% of the total number of cases and an additional 14% were added when a symptom duration cut-off of ≤10 days was used. However, among individuals aged >5 years, only 39% of influenza positive cases had a duration of symptoms of ≤7 days and an additional 27% were added when a symptom duration cut-off of ≤10 days was used [18].

Most of the danger signs included in the pre-2011 WHO case definition have low sensitivities but high specificities. The exclusion of these danger signs from the 2014 WHO case definition did not significantly affect sensitivity and specificity, whilst improving simplicity [18]. This exclusion could have contributed to the creation of a simple and easily executable case definition since fewer criterions for surveillance screening are required. Our study supports this change.

Our analysis showed that the 2014 WHO case definition with fever ≥38°C only had a moderate sensitivity but high specificity in both HIV-uninfected and HIV-infected children compared to the higher sensitivity and lower specificity of the 2014 WHO case definition with any fever, making the inclusion of history of fever desirable if higher sensitivity is preferred[19]. On the contrary, an inclusion of measured fever is advised in circumstances where higher specificity is desired [16].

Our study has potential limitations that warrant discussion. The data that we used for the study did not capture information on the level of immunosuppression or use of antiretroviral treatment (ART) among the children that were HIV infected. Some of the HIV-infected children are likely to have been on ART, and this could have made their clinical presentation more similar to those of HIV-uninfected children. It is therefore possible that the observed similarity in the performance of the 2014 WHO SARI case definition for the detection of influenza virus infections among HIV-infected and HIV-uninfected children could have resulted from the beneficial effect of ART in HIV-infected children. In addition, since the number of HIV-infected children with influenza was low, failure to detect an association with symptoms could have been due to lack of statistical power.

## Conclusion

In conclusion, the pre-2011 and 2014 WHO SARI case definitions have similar sensitivity and specificity in both HIV-infected and HIV-uninfected children, but the latter is simpler and easier to apply. Importantly, the 2014 WHO case definition performed similarly in both HIV-infected and HIV-uninfected children, making it suitable for use in a high HIV prevalence setting. Our findings support the adoption of the 2014 WHO case definition for children aged <5 years irrespective of HIV infection status. Our study was limited to individuals <5 years, we therefore recommend the same analysis among HIV-infected and HIV-uninfected individuals aged ≥5 years.
